# Economic Deprivation and Its Effects on Childhood Conduct Problems: The Mediating Role of Family Stress and Investment Factors

**DOI:** 10.3389/fpsyg.2017.01580

**Published:** 2017-09-13

**Authors:** Edward M. Sosu, Peter Schmidt

**Affiliations:** ^1^School of Education, University of Strathclyde Glasgow, United Kingdom; ^2^Justus Liebig University Giessen Giessen, Germany

**Keywords:** conduct problems, economic deprivation, poverty, family stress model, investment model, cognitive ability, preschool

## Abstract

This study investigated the mechanisms by which experiences of poverty influence the trajectory of conduct problems among preschool children. Drawing on two theoretical perspectives, we focused on family stress (stress and harsh discipline) and investment variables (educational investment, nutrition, and cognitive ability) as key mediators. Structural equation modeling techniques with prospective longitudinal data from the Growing Up in Scotland survey (*N* = 3,375) were used. Economic deprivation measured around the first birthday of the sample children had both direct and indirect effects on conduct problems across time (ages 4, 5, and 6). In line with the family stress hypothesis, higher levels of childhood poverty predicted conduct problems across time through increased parental stress and punitive discipline. Consistent with the investment model, childhood deprivation was associated with higher levels of conduct problems via educational investment and cognitive ability. The study extends previous knowledge on the mechanisms of this effect by demonstrating that cognitive ability is a key mediator between poverty and the trajectory of childhood conduct problems. This suggests that interventions aimed at reducing child conduct problems should be expanded to include factors that compromise parenting as well as improve child cognitive ability.

## Introduction

Strong associations exist between poverty in early childhood and problem behavior in later life (e.g., Dearing et al., 2006; Sun et al., 2015; Mazza et al., 2016). While not all children living in economic hardship go on to display conduct problems, a disproportionately high number of children with conduct problems tend to come from families living in poverty (Boe et al., 2012). Evidence from longitudinal studies (e.g., Kiernan and Huerta, 2008; Rijlaarsdam et al., 2013) have identified poverty in early childhood as a risk antecedent to problem behavior across the lifespan. Additionally, experimental and longitudinal findings demonstrate that changes in family income directly lead to changes in child conduct problems (Costello et al., 2003; Morris and Gennetian, 2003; Votruba-Drzal, 2006). While these findings suggest a causal link between poverty and conduct problems, the mechanism by which economic deprivation leads to conduct problems remains unclear.

### Poverty and conduct problems: theories on the mechanisms of effect

Two theoretical perspectives that have been extensively deployed to explain this mechanism are the family stress model and the investment model (Mayer, 1997; Conger et al., 2010). Both theories posit an indirect effect of poverty on childhood conduct problems. Boss et al. (2017, p. 4) defined family stress as “a disturbance in the study state of the family system.” Such a disturbance may be due to external factors such as, unemployment or internal factors such as, divorce. Others (e.g., McCubbin et al., 1980) have conceptualized family stress as the response of a family to distressing life events and tensions generated by these events. According to the family stress model, economic deprivation induces psychological distresses such as, depression, anxiety, and parental stress, due to the strain of having fewer resources available for day-to-day living. Such stressors are associated with frustration and aggressive interactions (Berkowitz, 1989) which in turn lead parents to adopt punitive or unresponsive parenting styles with consequences for childhood conduct trajectories (Conger et al., 2010). Support for this model comes from studies demonstrating a link between poverty, parental psychological distress, punitive discipline, and conduct problems (Gershoff et al., 2007; Kiernan and Huerta, 2008; Rijlaarsdam et al., 2013).

Family investment on the other hand is defined as the amount of money parents put into purchasing quality education, nutrition, health, good neighborhood, and other inputs that improves a child's future well-being (Mayer, 2002). This investment is determined by a family's income. The investment model proposes that poverty restricts parents' ability to provide enriching educational experiences and services, as well as sufficiently nutritious diets. This in turn leads to lower cognitive abilities with potential consequences for other developmental domains (Mayer, 1997; Conger et al., 2010). Economic deprivation has been found to longitudinally predict low educational investment and consequently cognitive abilities (Kiernan and Huerta, 2008; Sun et al., 2015). Additionally, changes in parental economic circumstances predict investment in nutritious diets (Skafida and Treanor, 2014), and childhood malnutrition has been linked to low cognitive ability and conduct problems in adolescence (Galler et al., 2012).

Recent extensive reviews of the application of the family stress and investment models show that very few studies (e.g., Guo and Harris, 2000; Yeung et al., 2002) have simultaneously integrated elements from the two models in understanding a single developmental outcome such as, conduct problems (Conger et al., 2010; Shaw and Shelleby, 2014). Most studies employing both models in a single study have used them to explain different outcomes, that is, the family stress model being used to explain behavioral outcomes and the investment model to explain cognitive outcomes (e.g., Gershoff et al., 2007; Kiernan and Huerta, 2008). Where both models have been used to explore pathways from poverty to conduct problems (e.g., Linver et al., 2002; Rijlaarsdam et al., 2013), these were not directly predicted from the main consequence of low investment, that is, cognitive ability. It is well established that poverty directly stunts the development of those cognitive competences (e.g., executive function, language, working memory, and decision making) that underpin children's emotional and self-regulatory responses (Noble et al., 2005; Farah et al., 2006), mechanisms that are directly linked to conduct problems or tendency to take on prosocial roles such as, standing up to bullies (Belacchi and Farina, 2010; Montroy et al., 2014). Concurrent association studies have also found that cognitive ability predicts conduct problems (e.g., Bellanti and Bierman, 2000). Further, Galler et al. (2012) found that the effect of childhood malnutrition on conduct problems in adolescence was mediated by cognitive ability. It is therefore no surprise that interventions aimed at improving cognitive ability and underpinning processes such as, emotional regulation also lead to improvements in child conduct problems or gains in prosocial behavior, and those aimed at improving behavior result in cognitive benefits (Lunkenheimer et al., 2008; Scott et al., 2010; Ornaghi et al., 2017). In other words, an investment pathway from poverty to conduct problems should include cognitive ability as a key mediator.

Closely linked to the above are calls to explore other pathways between poverty and childhood outcomes within the context of these models. For instance, Shaw and Shelleby (2014) argued for the testing of a direct path between parental distress and childhood conduct problems, beyond the indirect effect through parenting because associations between parental distress and conduct problems may depend on factors other than compromised parenting. One argument is that maternal psychological distress can have direct effects on childhood conduct problems through heritability of negative traits linked to conduct problems during pregnancy (Goldsmith et al., 1997; Kim-Cohen et al., 2005). According to Shaw and Shelleby (2014), parental stress during pregnancy can induce neuroendocrine alterations which in turn lead to development of negative traits, such as, irritability, associated with conduct problems. Other researchers have documented direct effects between economic deprivation and conduct problems (Kiernan and Huerta, 2008), suggesting that the effect of poverty may not be completely mediated by family stress and investment variables.

Further, researchers have critiqued the limited use of longitudinal data in testing these models among children (Conger et al., 2010; Shaw and Shelleby, 2014). We came across only one study that used data matching the temporal ordering of predictors, mediators and outcome variables (i.e., Rijlaarsdam et al., 2013). Additionally, only one recent longitudinal study using the family stress model (e.g., Mazza et al., 2016) have examined the effect of deprivation on conduct problems over time, and we are not aware of any study combining both stress and investment mediators to examine conduct problems over time.

### Focus of the current study

**Figure 1:**
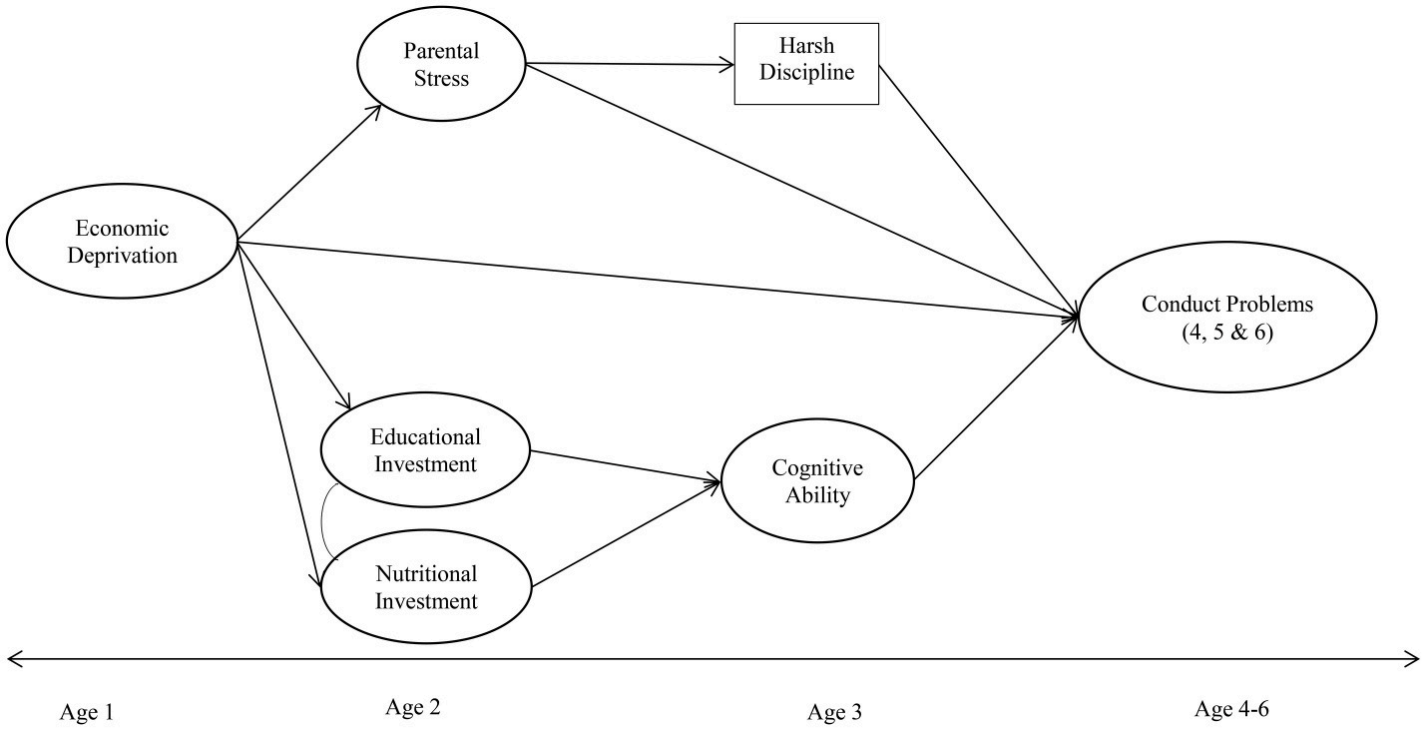
An integrated family stress and investment model for childhood conduct problems. Only latent factors are shown here for purposes of simplicity. All latent constructs (except Harsh Discipline) were measured with multiple indicators. Please see section on methodology and Table 1 for number of items used to measure each construct and model specification.

The current longitudinal prospective study was conceptualized to examine pathways by which experiences of economic deprivation in early childhood influence the trajectory of conduct problems during the preschool years. We focused on the preschool years because familial economic circumstances during the early years are crucial for development (Votruba-Drzal, 2006). At this age, children are highly dependent on their families and therefore more sensitive to contextual influences such as, poverty (e.g., Bronfenbrenner, 1977). To achieve our research goal, we integrated elements from both the family stress model and the investment model. We simultaneously examined the extent to which resultant family stress variables (stress and harsh parenting) and investment variables (educational investment, nutrition and cognitive ability) mediate the relationship between economic hardship measured when children were 10 months old, and trajectory of conduct problems from ages 4 to 6. We hypothesized the following (Figure 1):

1. Parental economic deprivation will have a *direct effect* on the trajectory of conduct problems (i.e., higher conduct problems across ages 4, 5, and 6).
2. Parental economic deprivation will have an *indirect effect* on the trajectory of conduct problems via increased parental psychological distress and punitive parenting.
3. Parental economic deprivation will have an *indirect effect* on the trajectory of conduct problems via low educational investment, poor nutrition, and low child cognitive ability.

Considering that early experiences of poverty can lead to childhood conduct problems, and childhood conduct problems are risk antecedents for poverty in adulthood (Fergusson et al., 2005), we envisage the findings to offer information on effective strategies for prevention and intervention that might break this cycle.

## Methods

### Data and participants

Data from the Growing Up in Scotland Survey (GUS), a national longitudinal survey, was used for this study. To ensure a nationally representative sample, a multi-stage stratified random sampling technique of all eligible children within a cohort year was employed. Data were obtained annually through face-to-face interviews with the child's main caregiver (mostly the child's mother, 95.5% of respondents). A detailed description of the sampling procedure and method of data collection is available on the GUS webpage and in the official user guide (ScotCen Social Research, 2013).

For the current study, data from wave 1 (obtained in 2005/06) to wave 6 (obtained in 2010/11) of the first Birth Cohort survey were used. Wave 1 data was collected when the children were 10.5 months old. Subsequent waves were obtained at 22, 34.5, 46, 58, and 70 months, respectively. A total of 5,217 children born between June 2004 and May 2005 were recruited for the initial survey in wave 1. Of these, 3,375 participants who responded to all six waves of data collection were retained for analysis. This represents 94.2% of all eligible respondents (those who completed all previous 5 waves) and 64.7% of all Wave 1 cases. To overcome limitations of sample attrition, birth cohort longitudinal weights were used in the analyses to help attenuate biases associated with non-random attrition (ScotCen Social Research, 2013). The sample children consisted of 51.3% male and 48.7% female. Ethnicity of the cohort children as designated in the GUS dataset was 96.5% “White” and 3.5% “Other ethnic background.”

### Ethical approval

The GUS study was subject to a medical ethical review and received approval from the Scotland “A” MREC committee (application reference: 04/M RE 1 0/59). Approval for the use of the data for this study was obtained through the UK Data Service.

### Measures

A strategy was adopted to select variables sequentially to reflect the hypothesized pathways (Figure 1). The dependent variable, *conduct problems* was measured at ages 4, 5, and 6. The predictor variables, *parental economic deprivation* was measured at age 1, *psychological distress, educational investment*, and *nutrition* at age 2, *child cognitive ability* at age 3, *harsh discipline* although measured at age 4 reflected parental behavior at age 3. Descriptive statistics are represented in Table 1.

**Table 1:** Descriptive statistics (range, means, standard deviation, percentages) and item level standardized factor loadings **(**β**)** of sample, predictor and dependent variables (*n* = 3,375).

	**Range**	**Mean**	**SD**	**β**	**%**
**ECONOMIC DEPRIVATION(T1)**					
Income quintiles	1–5	3.06	1.42	0.83	–
Subjective poverty	1–5	2.79	0.87	0.50	–
**STRESS(T2)**					
Upset	1–4	1.64	0.71	0.74	–
Difficult to relax	1–4	1.83	0.79	0.71	–
Irritable	1–4	1.73	0.68	0.72	–
**EDUCATIONAL INVESTMENT(T2)^b^**					
Look at books	1–7	0.81	1.76	0.56	–
Nursery rhymes	1–7	2.02	2.66	0.47	–
Recognizing shapes, letters, numbers	1–7	3.77	2.88	0.37	–
**NUTRITIONAL INVESTMENT(T2)^b^**					
Vegetables	1–5	2.26	0.75	0.48	–
Fruits	1–5	1.88	0.79	0.63	–
**COGNITIVE ABILITY(T3)**					
Naming vocabulary	20–80	47.95	12.37	0.69	–
Picture similarity	20–80	50.25	10.58	0.57	–
Harsh Discipline (T3)	0–1	0.39	0.49	–	–
**CONDUCT PROBLEMS (T4, *T5*, T6)^a^**					
Tantrums	0–2	0.92, *0.79*, 0.68	0.72, *0.70*, 0.69	0.66, *0.71*, 0.74	–
Obedient	0–2	0.68, *0.59*, 0.56	0.52, *0.57*, 0.58	0.54, *0.57*, 0.60	–
Fights	0–2	0.11, *0.10*, 0.09	0.33, 0.34, 0.31	0.65, *0.65*, 0.70	–
Lies	0–2	0.20, *0.22*, 0.22	0.43, *0.45*, 0.45	0.47, *0.52*, 0.56	–
Steals	0–2	0.03, *0.03*, 0.03	0.19, *0.21*, 0.20	0.50, *0.50*, 0.56	–
Gender (male)	–	–	–	–	51.3
Ethnicity (White)	–	–	–	–	96.5

a The values represent range, mean, standard deviation (SD) and factor loadings of items used to measure conduct problems **(**β**)** across waves 4, 5, and 6 of data collection respectively. Means, standard deviations, factor loadings and percentages are based on a weighted sample;

b *higher scores represent low educational and nutritional investment*.

### Conduct problems

Child conduct problems were measured using five items from Goodman's (1997) Strength and Difficulties Questionnaire (SDQ). The SDQ has good structural, concurrent, discriminant, convergent, and predictive validity (e.g., Kersten et al., 2016), and it is measurement invariant across time (Sosu and Schmidt, 2017). The instrument was administered to parents when their children were just under 4, 5, and 6 years of age. Parents were asked to indicate the extent to which the sample child engages in five specific behaviors (tantrums; fights; lies; steals; and obedient which was reverse coded). The five items were measured on a 3-point scale (0 = Not true; 1 = Somewhat true; 2 = Certainly). Due to the polytomous nature of the conduct problem's response scale, we explored reliability within a structural equation modeling framework (Brown, 2015). More specifically, we tested for longitudinal measurement invariance to enable us judge whether the scale was configural, metric, or scalar invariant over time. Such information is crucial for longitudinal studies as it tells us whether respondents' understanding of items and constructs measured by an instrument remain the same across time. Finding from this analysis suggest the conduct problem scale is reliable (see result section reporting on item reliability and measurement invariance).

### Parental economic deprivation

Two items, equivalized income and subjective poverty, were used to measure parental economic deprivation. These were obtained when children were about 10 months of age. To measure *equivalised income*, parents were first asked to select from a range of 17 income bands (1–less than £3,999 to 17–56,000 or more), the amount that best represented their family income before tax including all state benefits and interests. All income bands between the minimum and maximum described above had a range of about £2,000 (i.e., 4,000–5,999; 6,000–7,999, etc.). The figures were then equalized by adjusting for differences in household size and composition (see e.g., Scottish Government, 2009; Bradshaw et al., 2015) and converted into quintiles with a range from 1 (>£33,571) to 5 (<£8,410). *Subjective poverty* was measured through perceived economic pressure. Parents were asked to rate how they feel about managing on their present income. Responses were on a 5-point scale ranging from 1 (Living very comfortably on present income) to 5 (Finding it very difficult on present income). Higher scores on both items represent a higher level of deprivation.

### Nutrition

Nutrition was measured using two items obtained from parents when the children were 2 years old. Parents were asked to indicate how many different types of fresh, frozen or tinned fruit and vegetable their child eats on a typical day. Responses were on a 5-point scale (0–More than five to 4–None), with higher scores indicating poorer nutrition. These two items were chosen in line with previous studies indicating significant associations between income deprivation and consumption of fruits and vegetables (Skafida and Treanor, 2014).

### Educational investment

Educational investment was measured when children were 2 year's old with three items. Parents were asked to respond to the question: “Can you tell me on how many days in the last week *childname* has done each of the following things either on his own or with someone else? By “the last week,” I mean the last 7 days.” The items were: looking at books or read stories; reciting nursery rhymes; and recognizing letters, words, numbers, or shapes. Responses were coded from 0 to 7 so that higher scores represent low educational investment. These items represent proximal measures of educational investment and have been used in previous studies (e.g., Guo and Harris, 2000; Yeung et al., 2002). While it can be argued that the measure may be child-driven, it was obtained when the cohort children were just 2 years of age, a time when parents are more likely to be the ones shaping their children's interests.

### Parental psychological stress

Parental psychological stress was measured when children were 2 years old using three selected items from the Depression, Anxiety, and Stress Scale (Lovibond and Lovibond, 1995). The complete scale has well established psychometric properties (Henry and Crawford, 2005). Participants were asked to indicate how much the following statements applied to them over the past week: “I found myself getting upset rather easily,” “I found it difficult to relax” and “I found that I was very irritable” measured on a 4 point scale (1–Did not apply to me at all to 4–Applied to me very much or most of the time).

### Child cognitive ability

Cognitive ability was measured at age 3 using the naming vocabulary and picture similarities subtests of the British Ability Scales Second Edition (BAS II; Elliott et al., 1997). Studies indicate that the BAS has a sound theoretical underpinning, possesses good psychometric properties and is age appropriate compared to other available tests (Hill, 2005). Naming vocabulary assesses expressive language ability and development, while picture similarities assess problem solving and reasoning ability. For the current study, T-scores derived from normative scores (with a range from 20 to 80, and a mean 50) for both the naming vocabulary and picture superiority scales were used. Items were recoded so that higher scores indicating low cognitive ability.

### Harsh discipline

Harsh discipline was measured using parental response to one question when the children were 4 years old. Participants were asked to indicate whether they have ever used smacking with the named child in the previous year (corresponding to age 3) during which the question was not asked. Response to this item was dummy coded (No—0; Yes—1).

### Analytic procedure

Analysis was undertaken using longitudinal structural equation modeling (SEM). First, longitudinal measurement invariance of the conduct problems scale was tested to ascertain whether the conduct problems scale was measuring the same construct across time (Widaman et al., 2010). Measurement invariance was sequentially examined by testing for configural, metric, and scalar invariance over time (Davidov et al., 2011). Second, an unconditional latent growth model (LGM) was estimated to evaluate the trajectory of conduct problems over time. Unconditional models do not include predictors of change (Meredith and Tisak, 1990; Davidov et al., 2011). LGMs generally estimate an intercept mean (i.e., average conduct problems at age 4), intercept variance (i.e., individual differences in conduct problem at age 4), slope mean (i.e., rate of change in conduct problem from ages 4 to 6 for the entire sample), and slope variance (i.e., individual differences in the rate of change). Since our analysis was a multiple indicator LGM with ordinal items, the mean of the intercept (i.e., average conduct problems score at age 4) is not estimated due to model specification procedures (see Muthén and Muthén, 2012 for detailed explanation). Third, following outcomes of the unconditional LGM, models hypothesizing both direct and indirect effects of economic deprivation via family stress and investment mediators on trajectory of conduct problems (i.e., across ages 4, 5, and 6) were tested.

To determine evidence of indirect effects, we examined the statistical significance of direct paths linking parental economic deprivation, associated mediators and outcomes in each hypothesized mediation process, as well as confidence intervals of indirect paths (MacKinnon et al., 2007; Kenny, 2016). All variables (predictor, mediator, and outcome) except for harsh discipline were modeled as latent constructs.

### Model estimation, attrition, and missing data

Since, items underpinning the conduct problems scale were measured on an ordinal (polytomous) scale, the weighted least squares means-variance (WLSMV) estimation procedure which yields more accurate parameter estimates, and standard errors when ordinal level data are modeled was used (Byrne, 2012). All analyses were undertaken using Mplus 7.4.

A key problem with all longitudinal studies is attrition. Within the GUS data, attrition analysis showed that those who are unemployed, live in large urban areas, less likely to indicate their income at a previous sweep, and younger parents were more likely to drop out of the study (ScotCen Social Research, 2013). The GUS data includes longitudinal weights generated using sociodemographic characteristics associated with non-response (ScotCen Social Research, 2013). These weights were taken into account in the computation of model fit indices and parameter estimates in our analysis. With respect to missingness, there was negligible missing data on items used to measure conduct problems over time (average of 1.3%, 1.5%, and 0.98% across age 4, 5, and 6, respectively). Average missing data for covariates was equally small (2.3%), with a range from 0 (no items missing for nutrition) to 8.9% (income quintiles). According to Asparouhov and Muthén (2010), the WLSMV approach for treatment of missing data implemented in Mplus produces unbiased estimates when the amount of missing data is not substantial and the model includes covariates that predict missingness.

### Model evaluation

Goodness of fit was evaluated using the Tucker-Lewis index (TLI) and comparative fit index (CFI) with values >0.90 and 0.95 indicative of “adequate” and “good” fit respectively, and root mean square error of approximation (RMSEA) values lower than 0.05 as evidence of good fit (Hu and Bentler, 1999; Marsh et al., 2004). Nested models are tested when evaluating measurement invariance. Although the chi-square difference test is recommended for evaluating such models, it is sensitive to marginal differences and performs poorly against other indices such as, changes in CFI and RMSEA (Cheung and Rensvold, 2002; Chen, 2007; Little, 2013). Thus, we used changes in CFI of >0.01 and RMSEA of > 0.015, as well as overall fit of each model to determine measurement invariance (Chen, 2007; Little, 2013). Specifically, a model was invariant if at least one of the indices was within the cut-off benchmark and the overall model was a good fit. Finally, to determine the strength and the practical utility of our indirect and total effects, we evaluated the effect size of our standardized coefficients with values of 0.01, 0.09, and 0.25 representing small, medium and large effects respectively. These thresholds represent appropriate benchmarks for determining small, medium, and large effects when reporting completely standardized indirect effects (Cohen, 1988; Preacher and Kelley, 2011; Kenny, 2016).

## Results

### Descriptive statistics and item reliability

Detailed descriptive statistics for all variables are represented in Table 1. Goodman (1997) provided the following cut-off points for the composite scale of conduct problems: Normal (0 to 2), Borderline (3), and Abnormal (4 to 10). Consistent with previous studies (e.g., Goodman, 1997), the proportion of children in the current sample who fell into the abnormal score range were 14% at age four, 12% at age five, and 10% at age six. Borderline conduct problems were 17, 14, and 12% across ages four to six respectively.

Using the 2005/06 income threshold, that is, at the time of data collection, for defining who is living in poverty in the United Kingdom (Scottish Government, 2009), all respondents in the bottom income quintile (22%) would have been living in poverty (i.e., had income below 60% of the UK median). Taking into account the proportion of respondents who reported finding it difficult or very difficult managing on their current income (18%), it can be concluded that about 18 to 22% of the sample children were living in households experiencing economic deprivation. This figure is similar to the proportion of children living in relative poverty (21%) in Scotland at the time of data collection (Scottish Government, 2009).

Average item level standardized factor loadings based on outcome of structural equation models (Table 1) for conduct problems (0.56, 0.59, 0.64, at ages four, five and six respectively), economic deprivation (0.66), stress (0.72), poor nutrition (0.56), low educational investment (0.47), and low cognitive ability (0.63) suggests that, on the whole, the items used to measure these latent constructs were both valid and reliable (Brown, 2015).

Preliminary analysis exploring gender differences on our predictor, mediator, and outcome variables were undertaken since it is well established that boys generally demonstrate higher conduct problems than girls (Rutter et al., 2003). Results (Table 2) indicate that boys had significantly higher conduct problem scores than girls across the three time points. Additionally, there was greater parental investment in the education of girls than boys, and girls obtained significantly higher cognitive ability scores. Significant associations were also observed between gender and use of harsh discipline, with parents reporting greater use of smacking with boys than girls. No significant gender differences were observed for economic deprivation, parental stress, or nutritional investment.

**Table 2:** Gender differences on predictor, mediator and outcome variables.

**Variables**	**Gender**		
	**Male (*Mean, SD*)**	**Female (*Mean, SD*)**	***p***
Conduct problem^a^	1.90 (1.15)	1.39 (1.38)	0.001
Economic deprivation	2.92 (0.97)	2.89 (0.96)	0.414
Stress	2.23 (1.81)	2.18 (1.77)	0.318
Educational investment	5.14 (1.64)	5.64 (1.30)	0.001
Nutritional investment	1.92 (0.64)	1.95 (0.60)	0.242
Cognitive ability	49.19 (9.90)	52.78 (9.04)	0.001
Harsh discipline (% smacked)^b^	19%	14%	0.001

a *The mean for conduct problem presented in the table is average across the 3 time points for purposes of parsimony. The analysis was undertaken for each individual time point (ages 4, 5 and 6) and these were all significant*.

b *Harsh discipline analyzed using chi-square test*.

### Longitudinal measurement invariance of the conduct problems scale

Results from the first analysis revealed that the conduct problems scale was configural, metric, and scalar invariant over time (Table 3). A comparison between the configural and metric invariance model using our stated criteria (ΔRMSEA and ΔCFI) suggests that there was no significant deterioration in the model. With regards to metric and scalar models, the ΔCFI suggests an absence of invariance, while the ΔRMSEA indicate the scale was scalar invariant. Since our examination of modification indices and other parameter estimates did not show any significant form of local misfit, and the overall model had a good fit, we concluded that the conduct problems scale was scalar invariant in line with our stated criteria. Furthermore, it measured the same construct across the three measurement periods and it is legitimate to compare latent means over time.

**Table 3:** Fit indices for measurement invariance, unconditional latent growth model (LGM), and trajectory model.

**Model**	**X^2^**	**df**	**CFI**	**TLI**	**RMSEA [90% CI]**	**ΔCFI**	**ΔRMSEA**	**ΔWLSMV X^2^ (Δ*df*)**
**MEASUREMENT INVARIANCE**								
Configural	296.14^***^	72	0.98	0.97	0.030 [0.027–0.034]	–	–	–
Metric	267.04^***^	80	0.98	0.98	0.026 [0.023–0.030]	0.003	0.004	4.409 (8)^ns^
Scalar	567.86^***^	100	0.96	0.96	0.037 [0.034–0.040]	0.025	0.011	388.26(20)^***^
Unconditional LGM	320.94^***^	89	0.98	0.98	0.028 [0.025–0.031]	–		–
**TRAJECTORY MODEL**								
Conduct Age 4	311.11^***^	124	0.96	0.95	0.021 [0.018–0.024]			–
Conduct Age 5	319.41^***^	124	0.96	0.95	0.022 [0.019–0.025]			
Conduct Age 6	312.37^***^	124	0.96	0.95	0.021 [0.018–0.024]			

*X^2^, chi-square; df, degree of freedom; CFI, comparative fit index; TLI, Tucker-Lewis Index; RMSEA, root mean square error of approximation with 95% confidence interval; ΔCFI, change in comparative fit index; ΔWLSMV X^2^, change in WLSMV chi-square using chi-square difference test; Δdf – change in degree of freedom*.

*** p < 0.001;

ns *nonsignificant*.

### Unconditional growth model: trajectory of conduct problems over time

In the second analysis we examined the trajectory of conduct problems without predictors. The findings indicate a good fit of the linear growth model (Table 3) with significant growth parameters (Table 4). The intercept variance (*b* = 0.33; *SE* = 0.033) suggested that children in this sample differed significantly on their initial level of conduct problems at age 4. The mean of the slope (*b* = −0.174; *SE* = 0.011) indicated that conduct problems decreased significantly during the preschool years. However, the slope variance was not significant (slope variance: *b* = 0.026; *SE* = 0.017), meaning that everyone declined at roughly the same rate over time. Additionally, covariance between intercept and slope was not significant (*b* = 0.031; *SE* = 0.018), suggesting that a child's initial level of conduct problems at age four was unrelated to the rate of change (between ages four and six). To ensure that the linear model provided the best description for the data, a nonlinear growth model was equally estimated. Since our data had only three data points, we modeled nonlinear growth (^*^, 1, 2) by freely estimating the first slope factor (Kamata et al., 2012; Nese, 2013). Assumptions of nonlinear growth were not supported (*b* = −0.25, *SE* = 0.23, *p* = 0.278).

**Table 4:** Results of the unconditional latent growth model for the trajectory of conduct problems.

**Growth parameter**	**Unconditional model estimates**		
	***b***	***SE***	***t***
Intercept^a^	–		–
Slope	−0.174	0.011	−16.008^***^
Var(intercept)	0.332	0.033	9.996^***^
Var(slope)	0.026	0.017	1.502
cov(intercept and slope)	0.031	0.018	1.771

a The mean of the intercept is not estimated in a multiple indicator LGM with ordinal items using WLSMV estimation. This is fixed at 1 to ensure model identification (Muthén and Muthén, 2012). Level of significance:

*** *p < 0.001*.

### Effect of economic deprivation on conduct problems across time—ages 4, 5, and 6

Since the slope variance from the unconditional model was not significant, we proceeded to investigate the effect of economic deprivation on trajectory of conduct problems by specifying generalized structural equation models rather than a conditional LGM which aims to predict variance of the slope (i.e., individual differences in the rate of change). Specifically, we tested three separate models (Figure 1) exploring the direct and indirect effects of economic deprivation on conduct problems across the three measurement time points (ages 4, 5, and 6). The model also included covariances between the two investment mediators. The hypothesized models had a good fit to the data (Table 3) and accounted for 27, 23, and 23% of the variance in conduct problems at ages 4, 5, and 6 respectively. The pattern of findings was similar across the three time points (Table 5).

**Table 5:** Standardized direct and indirect effects (via family stress and investment variables) of economic deprivation on trajectory of conduct problems.

**Direct and indirect pathways**	**Conduct age 4 *Std. Est(SE)***	**Conduct age 5 *Std. Est(SE)***	**Conduct age 6 *Std. Est(SE)***
**DIRECT EFFECTS**			
Econ dep→Conduct Problems (CP)	0.22 (0.04)^***^	0.19 (0.04)^***^	0.14 (0.04)^***^
Econ dep→stress	0.22 (0.03)^***^	0.22 (0.03)^***^	0.21 (0.03)^***^
Econ dep→educational investment	0.31 (0.04)^***^	0.30 (0.04)^***^	0.30 (0.04)^***^
Econ dep→nutritional investment	0.29 (0.04)^***^	0.29 (0.04)^***^	0.28 (0.04)^***^
Econ dep→cognitive ability	0.23 (0.04)^***^	0.23 (0.04)^***^	0.23 (0.04)^***^
Stress→harsh discipline	0.06 (0.02)^**^	0.06 (0.02)^**^	0.06 (0.02)^**^
Stress→CP	0.19 (0.03)^***^	0.20 (0.03)^***^	0.18 (0.03)^***^
Harsh discipline→CP	0.24 (0.03)^***^	0.17 (0.03)^***^	0.17 (0.02)^***^
Edu investment→cognitive ability	0.55 (0.05)^***^	0.54 (0.05)^***^	0.57 (0.05)^***^
Nutritional investment→cognitive ability	−0.002 (0.05)^ns^	0.00 (0.05)^ns^	−0.02(0.05)^ns^
Cognitive ability→CP	0.24 (0.04)^***^	0.24 (0.04)^***^	0.30 (0.04)^***^
**INDIRECT EFFECTS**			
Econ dep→stress→harsh discipline→CP	0.003 (0.001)^**^	0.002 (0.001)^**^	0.002 (0.001)^**^
Econ dep→stress→CP	0.04 (0.01)^***^	0.04 (0.01)^***^	0.04 (0.01)^***^
*Total Indirect Effect of Stress Pathway^a^*	0.043^***^	0.042^***^	0.042^***^
Econ dep→edu investment→cognitive ability→CP	0.04 (0.01)^***^	0.04 (0.01)^***^	0.05 (0.01)^***^
Econ dep→nutrition→cognitive ability→CP	0.00 (0.003)^ns^	0.00 (0.003)^ns^	−0.001 (0.004)^ns^
Econ dep→cognitive ability→CP	0.06 (0.01)^***^	0.06 (0.01)^***^	0.07 (0.02)^***^
*Total Indirect Effect of Investment Pathway^a^*	0.10^***^	0.10^***^	0.12^***^
*Total Indirect Effect – All Pathways*	0.14 (02)^***^	0.14 (02)^***^	0.16 (0.02)^***^
*Total Effect*	0.36 (0.03)^***^	0.33 (03)^***^	0.30 (0.03)^***^
*R*^2^	*27%*	*23%*	*23%*

Level of significance:

*** p < 0.001;

** p < 0.01;

ns *non-significant*.

*Econ dep, economic deprivation; edu investment, educational investment*.

a *Indirect effect of stress and investment pathways computed by adding together all significant associated indirect pathways*.

### Direct, indirect and total effects of economic hardship on trajectory of conduct problems

As shown in Table 5, economic deprivation when children were 10 months old had a significant direct effect on conduct problems at ages 4, 5, and 6 with higher levels of deprivation associated with higher conduct problems scores (*p* < 0.001). These results suggest that the effect of deprivation on conduct trajectory in the preschool years is not completely mediated by family stress and investment variables, supporting our first hypothesis.

In line with our hypothesized family stress model (Figure 1), economic deprivation had a significant direct effect on parental stress (*p* < 0.001), parental stress had a significant direct effect on parental discipline (*p* < 0.01), and parental discipline had a significant effect on conduct problems at ages 4, 5 and 6 (*p* < 0.001). Additionally, there was a significant direct effect from parental stress to conduct problems across all time points (*p* < 0.001). A significance test revealed two indirect effects consistent with the family stress mediators (deprivation→stress→discipline→conduct problems, *p* < 0.01; deprivation→stress→conduct problems, *p* < 0.001). That is, higher levels of deprivation resulted in higher levels of parental stress, which in turn led to greater use of harsh discipline and subsequently higher conduct problems at ages 4, 5, and 6. Additionally, the path via parental stress separately accounted for higher levels of conduct problems. The total indirect effect through the family stress model across the three time points (β = 0.04), indicate a medium effect size. These findings partially support our second hypothesis.

With respect to the hypothesis based on the investment model (Figure 1), economic deprivation was significantly associated with poorer nutrition (*p* < 0.001), lower educational investment (*p* < 0.001) and lower cognitive ability (*p* < 0.001). While lower educational investment had a significant direct effect on lower cognitive ability (*p* < 0.001), poorer nutrition was not significantly associated with cognitive ability (*p* > 0.10). Lower cognitive ability had a significant effect on conduct problems at ages 4, 5 and 6 (*p* < 0.001). Test of indirect effects via the investment mediators revealed two significant findings (deprivation→investment→cognitive ability→conduct problems *p* < 0.001; deprivation→cognitive ability→conduct problems *p* < 0.001). Thus, consistent with our hypothesis, experiences of economic deprivation influenced conduct problems across the preschool years via low educational investment and low cognitive ability, partially supporting our third hypothesis. The total indirect effect from the investment pathway was β = 0.10 to 0.12 across the three time points suggesting a medium effect. The total effect of all deprivation pathways on trajectory of conduct problems can be considered to be of large effect across all three time points (β = 0.30 to 0.36).

To check the robustness of our findings, we undertook follow-up analyses by controlling for the effect of gender on conduct problems, educational investment, cognitive ability, and harsh discipline due to the significant gender differences obtained in our preliminary analysis. The key model indices indicate significant fit for the age 4 (CFI = 0.96; TLI = 0.95), age 5 (CFI = 0.95; TLI = 0.94), and age 6 (CFI = 0.96; TLI = 0.95) models. Crucially, there were no changes in the significance of parameter estimates or direction of effects (full results available from the first author).

## Discussion

We used prospective longitudinal data to examine the mechanisms by which economic deprivation leads to conduct problems among preschool children. Consistent with the family stress model (Conger et al., 2010), economic deprivation measured around the first birthday of our sample children was indirectly associated with higher levels of conduct problems across the preschool years through effects on parental stress that increases the use of harsh discipline. Punitive parenting in turn led to higher conduct problems. We also found that elevated parental stress associated with poverty predicted increased levels of conduct problems, beyond effects through harsh discipline. This additional pathway concurs with Shaw and Shelleby (2014) hypothesis that associations between parental distress and conduct problems may depend on factors other than compromised parenting. One possible explanation is that there may have been maternal psychological distress during pregnancy which may have induced endocrine alterations that led to the transmission of negative traits linked to conduct problems (Goldsmith et al., 1997; Kim-Cohen et al., 2005). Another plausible explanation is that children living in poverty are equally exposed to stress. Thus, the direct effect from parental stress to child conduct problems may simply reflect the mediating role of child level stress in the pathway between poverty and conduct problems.

The investment pathway provided an equally valuable explanation for the association between poverty and conduct problems. As in previous studies (Mayer, 1997; Gershoff et al., 2007) economic deprivation restricted parental ability to invest in enriching educational experiences, which in turn led to lower cognitive ability in early childhood. Consistent with our hypothesis, cognitive ability predicted differences in conduct problems across ages 4, 5, and 6. Although poverty predicted nutritional investment in line with previous findings (Skafida and Treanor, 2014), the pathway from nutrition via cognitive ability was not significant, possibly due to the moderate association between the two investment variables (*r* = 0.44).

Overall, the above findings extend our theoretical understanding in that, an investment pathway from poverty to conduct problem should include cognitive ability as a key mediator in line with previous findings on the mediating role of cognitive ability on parental investment (Galler et al., 2012). Additionally, it demonstrates how the effect of childhood poverty on cognitive ability and conduct problems can create a cycle of poverty in adulthood. Children living in poverty are more likely to begin school with significant disadvantages that include lower cognitive ability and higher levels of conduct problems, factors that may make them lose substantial grounds in educational attainment to their peers (Masten et al., 2005; Montroy et al., 2014). A resultant poor educational outcome and increased conduct problems over time means fewer prospects and success in the labor market, thereby creating a cycle of poverty (Fergusson et al., 2005). Breaking this cycle will therefore require attention to both raising educational attainment and reducing conduct problems. Further research is however needed in order to understand the directionality and nature of the relationship between cognitive ability and conduct problems.

In contrast to the underlying assumption of both the family stress and investment models (Conger et al., 2010), the effect of poverty on childhood conduct problems was not completely mediated by stress and investment variables. Consistent with previous findings (Gershoff et al., 2007; Kiernan and Huerta, 2008), we found that economic deprivation directly influences conduct problems across the preschool years. However, not all studies have documented such direct effects and there is the suggestion that direct effects tend to be common when conduct problems are reported by caregivers other than by children or adolescents themselves (Sun et al., 2015). A more plausible explanation for our finding is that the effect of poverty may be mediated by other factors such as, childhood stress (Lupien et al., 2001; Evans and Kim, 2007). Future studies should therefore explore potential effects through child related variables such as, stress in addition to parental variables.

## Limitations of the study

Our study is limited by the fact that we mainly focused on the effect of poverty through psychosocial and investment mechanisms. It is likely that other yet to be explored pathways may add to the explanatory power of the integrated model. Evidence from biological theories suggests that conduct problems may be a result of genetic (e.g., Rhee and Waldman, 2002) and brain structure defects (e.g., Fairchild et al., 2013). However, it has been argued that possible genetic risks of childhood conduct problems may remain latent until children are exposed to adversities such as, economic hardship (e.g., Rutter et al., 2001; Costello et al., 2003). In other words, economic disadvantage may serve as the catalyst for genetic predisposition to conduct problems to become a reality. Future studies examining these mechanisms would shed light on the interaction between poverty and neuropsychological processes.

We are also aware that economic deprivation tends to vary over time rather than remain static. Thus, using economic deprivation when children were 10 months old may mask variability over time. However, compared to income in middle childhood, parental income during early childhood appears to be more influential on children's developmental trajectories (Votruba-Drzal, 2006). As evident in our findings, income measured when children were only 10 month old predicted a significant amount of variance in conduct trajectory. A subsequent analysis using cumulative measures of economic deprivation when the cohort children were about 1and 2 years of age did not alter our results. Finally, our study is correlational in nature and we were unable to model cross-lagged paths by adjusting for previous levels of predictor and mediator variables as these variables were not consistently available in our data set. Thus, our findings do not completely account for the directionality of effects and caution is needed when making causal attributions based solely on these findings. Although we used a sequential approach in selecting our predictor, mediator and outcome variables, experimental field studies and studies combining growth models with standard panel models offer future avenues for exploring causality of the underlying processes.

## Implications of the study

Despite the above limitations, our findings have significant implications with respect to identifying key areas of target for policy intervention. Firstly, helping families to overcome financial stress either through direct financial support or assistance to earn better income may help alleviate both parental stress and boost parental investment in education, key mediators of conduct problems. Approaches that increase family income do not only have positive effects on childhood behavior but also contribute to improvement in other outcomes including educational attainment (Costello et al., 2003; Morris and Gennetian, 2003; Votruba-Drzal, 2006). Although several countries including the UK provide social support for low income families, such support constitutes a minimal safety net and significant levels of poverty still exist (Scottish Government, 2016). Secondly, considering the mediating role of parental processes and cognitive ability, target domains for intervention need to be expanded to include factors that compromise parenting as well as improve cognitive ability for children. Evidence suggest that improvement in one domain can serve as a catalyst for changes in another (Lunkenheimer et al., 2008; Scott et al., 2010), and such multi-layered approaches may help break the cycle of poverty. Additionally, the significant direct effects observed between economic deprivation and conduct problems, as well as parental stress and conduct problems suggests that the effect of poverty on conduct problems is not exclusively a result of parental behaviors. As a result, interventions need to go beyond parenting programmes in a bid to reduce childhood conduct problems.

Finally, it is clear that poverty is a significant early risk antecedent for childhood conduct problems. Thus, policies that prioritize support for children at the very early stages before they begin formal schooling may prevent their behavior from getting worse and subsequently falling further behind their peers in educational achievement. While the initial cost of early intervention might be an immediate concern, this needs to be balanced against the fact that future costs associated with supporting children whose problems deteriorate by adulthood is equally substantial (Scott et al., 2001). Early interventions at the preschool stage also have a greater efficacy for reducing conduct problems than those for older children because childhood conduct problems and their associated parenting practices are more malleable during the early years (Olds, 2002; Reid et al., 2004). Additionally, as found in the current and previous studies (e.g., Mesman et al., 2009; Fanti and Henrich, 2010) conduct problems decrease as children grow older. Thus, early intervention should help quicken the pace of change for those at risk.

To conclude, the present study extended previous work on the exact mechanisms by which poverty leads to childhood conduct problems by demonstrating the role of cognitive ability as a key mediator between poverty and conduct problems. It is also only one of two studies using prospective longitudinal data matching temporal order of hypothesized variables, and the only one to examine trajectory of conduct problems across time. Interventions based on the integrated family stress and investment model may help improve conduct behavior for children from disadvantaged households, and by extension their future prospects.

## Author contributions

ES and PS contributed to the conceptualization of the study. ES undertook literature review. ES and PS undertook data analysis and contributed to the writing of the manuscript.

### Conflict of interest statement

The authors declare that the research was conducted in the absence of any commercial or financial relationships that could be construed as a potential conflict of interest. The reviewers and handling Editor declared their shared affiliation.

## References

[B1] Asparouhov T. Muthén B. (2010). Weighted Least Squares Estimation with Missing Data. Available online at: http://www.statmodel.com/download/GstrucMissingRevision.pdf

[B2] Belacchi C. Farina E. (2010). Prosocial/hostile roles and emotion comprehension in pre-schoolers. Aggress. Behav. 36, 371–389. 10.1002/ab.20361 20814973

[B3] Bellanti C. J. Bierman K. L. (2000). Disentangling the impact of low cognitive ability and inattention on social behavior and peer relationships. J. Clin. Child Psychol. 29, 66–75. 10.1207/S15374424jccp2901_7 10693033 PMC2767167

[B4] Berkowitz L. (1989). Frustration–aggression hypothesis: examination and reformulation. Psychol. Bull. 106, 59–73. 10.1037/0033-2909.106.1.59 2667009

[B5] Boe T. Overland S. Lundervold A. J. Hysing M. (2012). Socioeconomic status and children's mental health: results from the bergen child study. Soc. Psychiatry Psychiatr. Epidemiol. 47, 1557–1566. 10.1007/s00127-011-0462-9 22183690

[B6] Boss P. Bryant C. M. Mancini J. A. (2017). Family Stress Management: A Contextual Approach, 3rd Edn. Thousand Oaks, CA: Sage.

[B7] Bradshaw P. Knudsen L. Mabelis J. (2015). Growing Up in Scotland: The Circumstances and Experiences of 3-Year-Old Children Living in Scotland in 2007/08 and 2013. Edinburgh: The Scottish Government.

[B8] Bronfenbrenner U. (1977). Toward an experimental ecology of human development. Am. Psychol. 32, 515–531. 10.1037/0003-066X.32.7.513

[B9] Brown T. A. (2015). Confirmatory Factor Analysis for Applied Research, 2nd Edn. New York, NY: Guilford Press.

[B10] Byrne B. M. (2012). Structural Equation Modeling with Mplus: Basic Concepts, Applications, and Programming. New York, NY: Taylor & Francis Group.

[B11] Chen F. F. (2007). Sensitivity of goodness of fit indexes to lack of measurement invariance. Struct. Equ. Model. 14, 464–504. 10.1080/10705510701301834

[B12] Cheung G. W. Rensvold R. B. (2002). Evaluating goodness-of-fit indexes for testing measurement invariance. Struct. Equ. Model. 9, 233–255. 10.1207/S15328007SEM0902_5

[B13] Cohen J. (1988). Statistical Power Analysis for the Behavioral Sciences, 2nd Edn. New York, NY: Academic Press.

[B14] Conger R. D. Conger K. J. Martin M. J. (2010). Socioeconomic status, family processes and individual development. J. Marriage Fam. 72, 685–704. 10.1111/j.1741-3737.2010.00725.x 20676350 PMC2910915

[B15] Costello E. J. Compton S. N. Keeler G. Angold A. (2003). Relationships between poverty and psychopathology: a natural experiment. J. Am. Med. Assoc. 290, 2023–2029. 10.1001/jama.290.15.2023 14559956

[B16] Davidov E. Datler G. Schmidt P. Schwartz S. H. (2011). Testing the invariance of values in the Benelux countries with the European social survey: accounting for ordinality, in Cross-Cultural Analysis: Methods and Applications, eds Davidov E. Schmidt P. Billiet J. (New York, NY: Routledge), 149–172.

[B17] Dearing E. McCartney K. Taylor B. A. (2006). Within-child associations between family income and externalizing and internalizing problems. Dev. Psychol. 42, 237–252. 10.1037/0012-1649.42.2.237 16569163

[B18] Elliott C. D. Smith P. McCulloch K. (1997). The British Ability Scales II. Windsor, UK: NFER-NELSON Publishing Company.

[B19] Evans G. W. Kim P. (2007). Childhood poverty and health: cumulative risk exposure and stress dysregulation. Psychol. Sci. 18, 953–957. 10.1111/j.1467-9280.2007.02008.x 17958708

[B20] Fairchild G. Hagan C. C. Walsh N. D. Passamonti L. Calder A. J. Goodyer I. M. (2013). Brain structure abnormalities in adolescent girls with conduct disorder. J. Child Psychol. Psychiatry 54, 86–95. 10.1111/j.1469-7610.2012.02617.x 23082797 PMC3562487

[B21] Fanti K. S. Henrich C. C. (2010). Trajectories of pure and co-occurring internalizing and externalizing problems from age 2 to age 12: findings from the National Institute of Child Health and Human Development study of early child care. Dev. Psychol. 46, 1159–1175. 10.1037/a0020659 20822230

[B22] Farah M. J. Shera D. M. Savage J. H. Betancourt L. Giannetta J. M. Brodsky N. L.. (2006). Childhood poverty: specific associations with neurocognitive development. Brain Res. 1110, 166–174. 10.1016/j.brainres.2006.06.072 16879809

[B23] Fergusson D. M. Horwood L. J. Ridder E. M. (2005). Show me the child at seven: the consequences of conduct problems in childhood for psychosocial functioning in adulthood. J. Child Psychol. Psychiatry 46, 837–849. 10.1111/j.1469-7610.2004.00387.x 16033632

[B24] Galler J. R. Bryce C. P. Waber D. P. Hock R. S. Harrison R. Eaglesfield D.. (2012). Infant malnutrition predicts conduct problems in adolescents. Nutr. Neurosci. 15, 186–192. 10.1179/1476830512Y.0000000012 22584048 PMC3782078

[B25] Gershoff E. T. Aber J. L. Raver C. C. Lennon M. C. (2007). Income is not enough: incorporating material hardship into models of income associations with parenting and child development. Child Dev. 78, 70–95. 10.1111/j.1467-8624.2007.00986.x 17328694 PMC2835994

[B26] Goldsmith H. H. Buss K. A. Lemery K. S. (1997). Toddler and childhood temperament: expanded content, stronger genetic evidence, new evidence for the importance of environment. Dev. Psychol. 33, 891–905. 10.1037/0012-1649.33.6.891 9383612

[B27] Goodman R. (1997). The strengths and difficulties questionnaire: a research note. J. Child Psychol. Psychiatry 38, 581–586. 10.1111/j.1469-7610.1997.tb01545.x 9255702

[B28] Guo G. Harris K. M. (2000). The mechanisms mediating the effects of poverty on children's intellectual development. Demography 37, 431–447. 10.1353/dem.2000.0005 11086569

[B29] Henry J. D. Crawford J. R. (2005). The short-form version of the Depression Anxiety Stress Scales (DASS-21): construct validity and normative data in a large non-clinical sample. Br. J. Clin. Psychol. 44, 227–239. 10.1348/014466505X29657 16004657

[B30] Hill V. (2005). Through the past darkly: a review of the british ability scales second edition. Child Adolesc. Ment. Health 10, 87–98. 10.1111/j.1475-3588.2004.00123.x 32806801

[B31] Hu L. Bentler P. M. (1999). Cutoff criteria for fit indexes in covariance structure analysis: conventional criteria versus new alternatives. Struct. Equ. Model. 6, 1–55. 10.1080/10705519909540118

[B32] Kamata A. Nese J. F. T. Patarapichayatham C. Lai C. F. (2012). Modeling nonlinear growth with three data points: illustration with benchmarking data. Assess. Eff. Interv. 38, 105–116. 10.1177/1534508412457872

[B33] Kenny D. A. (2016). Mediation. Available online at: http://davidakenny.net/cm/mediate.htm#IE

[B34] Kersten P. Czuba K. McPherson K. Dudley M. Elder H. Tauroa R. (2016). A systematic review of evidence for the psychometric properties of the strengths and difficulties questionnaire. Int. J. Behav. Dev. 40, 64–75. 10.1177/0165025415570647

[B35] Kiernan K. E. Huerta M. C. (2008). Economic deprivation, maternal depression, parenting and children's cognitive and emotional development in early childhood. Br. J. Sociol. 59, 783–806. 10.1111/j.1468-4446.2008.00219.x 19035922

[B36] Kim-Cohen J. Moffitt T. E. Taylor A. Pawlby S. J. Caspi A. (2005). Maternal depression and child antisocial behavior: nature and nurture effects. Arch. Gen. Psychiatry 62, 173–181. 10.1001/archpsyc.62.2.173 15699294

[B37] Linver M. R. Brooks-Gunn J. Kohen D. E. (2002). Family processes as pathways from income to young children's development. Dev. Psychol. 38, 719–734. 10.1037/0012-1649.38.5.719 12220050

[B38] Little T. D. (2013). Longitudinal Structural Equation Modeling. New York, NY: Guilford Press.

[B39] Lovibond P. F. Lovibond S. H. (1995). The structure of negative emotional states: comparison of the Depression Anxiety Stress Scales (DASS) with the beck depression and anxiety inventories. Behav. Res. Ther. 33, 335–343. 10.1016/0005-7967(94)00075-U 7726811

[B40] Lunkenheimer E. S. Dishion T. J. Shaw D. S. Connell A. Gardner F. Wilson M. N. (2008). Collateral benefits of the family check up on early childhood school readiness: indirect effects of parents' positive behaviour support. Dev. Psychol. 44, 1737–1752. 10.1037/a0013858 18999335 PMC2769930

[B41] Lupien S. J. King S. Meaney M. J. McEwen B. S. (2001). Can poverty get under your skin? Basal cortisol levels and cognitive function in children from low and high socioeconomic status. Dev. Psychopathol. 13, 653–676. 10.1017/S0954579401003133 11523853

[B42] MacKinnon D. P. Fairchild A. J. Fritz M. S. (2007). Mediation analysis. Annu. Rev. Psychol. 58, 593–614. 10.1146/annurev.psych.58.110405.085542 16968208 PMC2819368

[B43] Marsh H. W. Hau K. Wen Z. (2004). In search of golden rules: comment on hypothesis testing approaches to setting cutoff values for fit indexes and dangers in overgeneralizing Hu and Bentler's (1999) findings. Struct. Equ. Model. 11, 320–341. 10.1207/s15328007sem1103_2

[B44] Masten A. S. Roisman G. I. Long J. D. Burt K. B. Obradovic J. Riley J. R. (2005). Developmental cascades: linking academic achievement and externalising and internalising symptoms over 20 years. Dev. Psychol. 41, 733–746. 10.1037/0012-1649.41.5.733 16173871

[B45] Mayer S. E. (1997). What Money Can't Buy: Family Income and Children's Life Chances. Cambridge, MA: Harvard University Press.

[B46] Mayer S. E. (2002). The Influence of Parental Income on Children's Outcomes. Wellington, NZ: Knowledge Management Group, Ministry of Social Development.

[B47] Mazza J. R. Pingault J.-B. Booij L. Boivin M. Tremblay R. Lambert J. (2016). Poverty and behavior problems during early childhood: the mediating role of maternal depression symptoms and parenting. Int. J. Behav. Dev. [Epub ahead of print]. 10.1177/0165025416657615

[B48] McCubbin H. I. Joy C. B. Cauble E. A. Comeau J. K. Patterson J. M. Needle R. H. (1980). Family stress and coping: a decade review. J. Marriage Fam. 42, 855–871. 10.2307/351829

[B49] Meredith W. M. Tisak J. (1990). Latent curve analysis. Psychometrika 55, 107–122. 10.1007/BF02294746

[B50] Mesman J. Stoel R. Bakermans-Kranenburg M. H. van IJzendoorn M. H. Juffer F. Koot H. M.. (2009). Predicting growth curves of early childhood externalising problems: differential susceptibility of children with difficult temperament. J. Abnorm. Child Psychol. 37, 625–636. 10.1007/s10802-009-9298-0 19184403

[B51] Montroy J. J. Bowles R. Skibbe L. E. Foster T. D. (2014). Social skills and problem behaviors as mediators of the relationship between behavioral self-regulation and academic achievement. Early Child Res. Q. 29, 289–309. 10.1016/j.ecresq.2014.03.002

[B52] Morris P. A. Gennetian L. A. (2003). Identifying the effects of income on children's development using experimental data. J. Marriage Fam. 65, 716–729. 10.1111/j.1741-3737.2003.00716.x

[B53] Muthén L. K. Muthén B. O. (2012). Mplus User's Guide, 7th Edn. Los Angeles, CA: Muthén & Muthén.

[B54] Nese J. F. T. (2013). Statistical Test for Latent Growth Nonlinearity with Three Time Points. National Center on Assessment and Accountability for Special Education (NCAASE) Available online at: http://ncaase.com/publications/in-briefs

[B55] Noble K. G. Norman M. F. Farah M. J. (2005). Neurocognitive correlates of socioeconomic status in kindergarten children. Dev. Sci. 8, 74–87. 10.1111/j.1467-7687.2005.00394.x 15647068

[B56] Olds D. (2002). Prenatal and infancy home visiting by nurses: from randomized trials to community replication. Prev. Sci. 3, 153–172. 10.1023/A:1019990432161 12387552

[B57] Ornaghi V. Brazzelli E. Grazzani I. Agliati A. Lucarelli M. (2017). Does training toddlers in emotion knowledge lead to changes in their prosocial and aggressive behavior toward peers at nursery? Early Educ. Dev. 28, 396–414. 10.1080/10409289.2016.1238674

[B58] Preacher K. J. Kelley K. (2011). Effect size measures for mediation models: quantitative strategies for communicating indirect effects. Psychol. Methods 16, 93–115. 10.1037/a0022658 21500915

[B59] Reid M. J. Webster-Stratton C. Baydar N. (2004). Halting the development of externalizing behaviors in head start children: the effects of parenting training. J. Clin. Child Adolesc. 3, 3279–3291. 10.1207/s15374424jccp3302_10 15136193

[B60] Rhee S. H. Waldman I. D. (2002). Genetic and environmental influences on antisocial behavior: a meta-analysis of twin and adoption studies. Psychol. Bull. 128, 490–529. 10.1037/0033-2909.128.3.490 12002699

[B61] Rijlaarsdam J. Stevens G. W. J. M. van der Ende J. Hofman A. Jaddoe V. W. V. Mackenbach J. P.. (2013). Economic disadvantage and young children's emotional and behavioral problems: mechanisms of risk. J. Abnorm. Child Psychol. 41, 125–137. 10.1007/s10802-012-9655-2 22736330 PMC3540352

[B62] Rutter M. Caspi A. Moffitt T. E. (2003). Using sex differences in psychopathology to study causal mechanisms: unifying issues and research strategies. J. Child Psychol. Psychiatry 44, 1092–1115. 10.1111/1469-7610.00194 14626453

[B63] Rutter M. Pickles A. Murray R. Eaves L. (2001). Testing hypotheses on specific environmental causal effects on behavior. Psychol. Bull. 127, 291–324. 10.1037/0033-2909.127.3.291 11393298

[B64] ScotCen Social Research (2013). Growing Up in Scotland: Cohort 1, Sweeps 1-6, 2005-2011. [data collection], 11th Edn. UK Data Service SN: 5760. Available online at: http://dx.doi.org/10.5255/UKDA-SN-5760-4

[B65] Scott S. Knapp M. Henderson J. Maughan B. (2001). Financial cost of social exclusion: follow up study of antisocial children into adulthood. BMJ 323:191. 10.1136/bmj.323.7306.191 11473907 PMC35269

[B66] Scott S. Sylva K. Doolan M. Price J. Jacobs B. Croo C.. (2010). Randomised controlled trial of parent groups for child antisocial behaviour targeting multiple risk factors: the SPOKES project. J. Child Psychol. Psychiatry 51, 48–57. 10.1111/j.1469-7610.2009.02127.x 19732250

[B67] Scottish Government (2009). Poverty and Income Inequality in Scotland: 2007/08. Available online at: http://www.gov.scot/Resource/Doc/933/0081018.pdf

[B68] Scottish Government (2016). Poverty and Income Inequality in Scotland: 2014/15. Available online at: http://www.gov.scot/Resource/0050/00502180.pdf

[B69] Shaw D. S. Shelleby E. C. (2014). Early-onset conduct problems: intersection of conduct problems and poverty. Annu. Rev. Clin. Psychol. 10, 503–528. 10.1146/annurev-clinpsy-032813-153650 24471370 PMC4194898

[B70] Skafida V. Treanor M. C. (2014). Do changes in objective and subjective family income predict change in children's diets over time? Unique insights using a longitudinal cohort study and fixed effects analysis. J. Epidemiol. Commun. Health 68, 534–541. 10.1136/jech-2013-203308 24441645

[B71] Sosu E. M. Schmidt P. (2017). Tracking emotional and behavioral changes in childhood: Does the Strength and Difficulties Questionnaire measure the same constructs across time? J. Psychoeduc. Assess. 35, 643–656. 10.1177/0734282916655503

[B72] Sun W. Li D. Zhang W. Bao Z. Wang Y. (2015). Family material hardship and Chinese adolescents' problem behaviors: a moderated mediation analysis. PLoS ONE 10:e0128024. 10.1371/journal.pone.0128024 26010256 PMC4444090

[B73] Votruba-Drzal E. (2006). Economic disparities in middle childhood development: does income matter? Dev. Psychol. 42, 1154–1167. 10.1037/0012-1649.42.6.1154 17087549

[B74] Widaman K. F. Ferrer E. Conger R. D. (2010). Factorial invariance within longitudinal structural equation models: measuring the same construct across time. Child Dev. Perspect. 4, 10–18. 10.1111/j.1750-8606.2009.00110.x 20369028 PMC2848495

[B75] Yeung W. J. Linver M. R. Brooks-Gunn J. (2002). How money matters for young children's development: parental investment and family processes. Child Dev. 73, 1861–1879. 10.1111/1467-8624.t01-1-00511 12487499

